# Supplementary material for: Polyscias filicifolia (Araliaceae) Hairy Roots with Antigenotoxic and Anti-Photogenotoxic Activity
Source: Molecules. 2021 Dec 29;27(1):186. doi: 10.3390/molecules27010186 (PMC8746452; doi:10.3390/molecules27010186)
Supplement: Supplementary file 1 [file molecules-27-00186-s001.zip › molecules-1466394-supplementary.pdf]

Polyscias filicifolia (Araliaceae) Hairy Roots with Antigenotoxic and Anti-photogenotoxic Activity

Anita Śliwińska[1], Ramona Figat[2*], Anna Zgadzaj[2], Beata Wilenska[3,4], Aleksandra Misicka[3,4], Grzegorz Nałęcz-Jawecki[2], Agnieszka Pietrosiuk[1] and Katarzyna Sykłowska-Baranek[1]

[1] Department of Pharmaceutical Biology and Medicinal Plant Biotechnology, Faculty of Pharmacy, Medical University of Warsaw, 1 Banacha St, 02-097 Warsaw, Poland; anita.sliwinska@wum.edu.pl (A.Ś.); katarzyna.syklowska-baranek@wum.edu.pl (K.S.B.); agnieszka.pietrosiuk@wum.edu.pl (A.P.)

[2] Department of Environmental Health Science, Medical University of Warsaw, Faculty of Pharmacy,
Banacha 1, 02-097Warsaw, Poland; rfigat@wum.edu.pl (R.F.); azgadzaj@wum.edu.pl (A.Z.); gnalecz@wum.edu.pl (G.N.J.)

[3] Faculty of Chemistry, University of Warsaw, 1 Pasteura St., 02-093 Warsaw, Poland; bwilenska@chem.uw.edu.pl (B.W.); misicka@chem.uw.edu.pl (A.M.)

[4] Biological and Chemical Research Centre, 101 Żwirki i Wigury St., 02-097 Warsaw, Poland

* Correspondence: e-mail@e-mail.com; rfigat@wum.edu.pl (R.F.); Tel.: +4822 5720740

Table S1. Effect of various media modifications on the growth of fresh, dry biomass and the K-1 line growth index of hairy roots of *Polyscias filicifolia*.

**Medium**	**Fresh biomass [mg]**	**Dry biomass [mg]**	**Growth index [%]**
DCR I with L- glutamine 500 mg/L, S-40 g/L	26.3 ± 1.14	2.37 ± 0.08	480.8 ± 23.63
WPM without growth regulators, S- 30 g/L	9.9 ± 0.46	0.75 ± 0.11	123.6 ± 12.32
WPM with L-glutamine 500 mg/L, S- 30g/L	11.2 ± 0.46	0.92 ± 0.02	149.3 ± 7.86
WPM without growth regulators, S- 40 g/L	12.4 ± 0.47	1.04 ± 0.01	177.10 ± 9.26
WPM with L-glutamine 500 mg/L, S- 40 g/L	14.4 ± 0.46	1.19 ± 0.14	220.5 ± 8.10

DCR I – DCR medium (Gupta and Durzan, 1985) modified by addition of L-glutamine 500 mg/L and with increased to 400 mg/L $MgSO_4$ x 7 H_2O (Syklowska-Baranek et al., 2009) and sucrose up to 40g/L.

WPM – Woody Plant Medium (Lloyd and McCown, 1981).

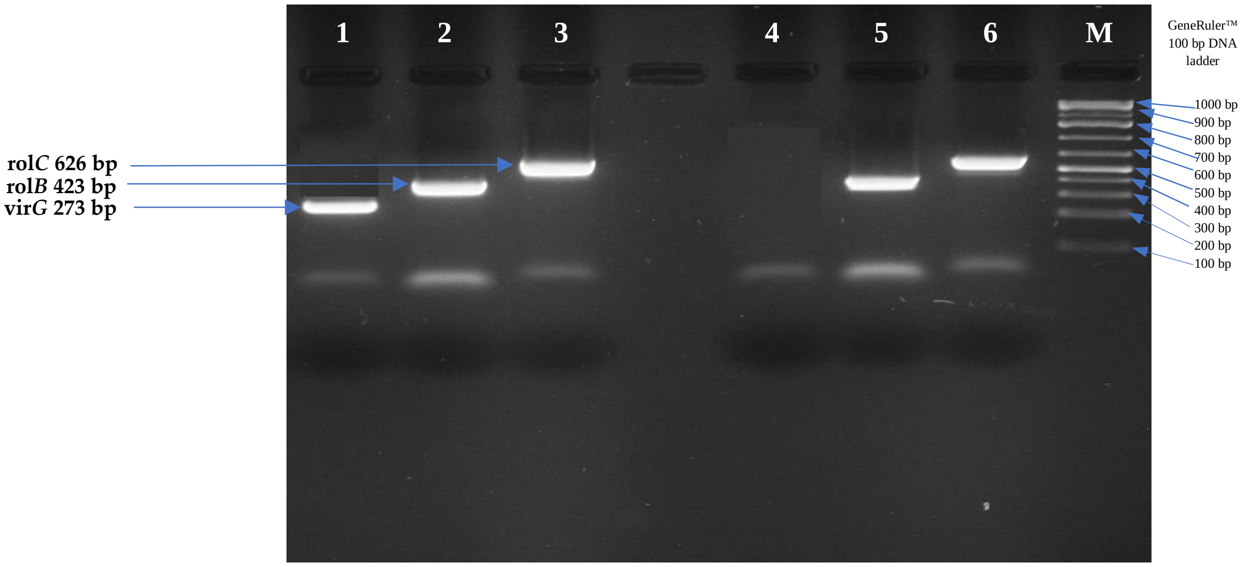

Figure S1. Confirmation of genetic transformation by PCR analysis of genomic DNA isolated from *Polyscias filicifolia* hairy roots of K-1 line:
M - molecular weight marker (GeneRuler™ 100 bp DNA ladder);
line 1 - *vir*G. line 2 - *rol*B (423 bp); line 3 - *rol*C (626 bp) of *Agrobacterium rhizogenes* 15834 Ri plasmid;
line 4: *vir*G in K-1 hairy roots; line 5: *rol*B in K-1 hairy roots; line 6: *rol*C in K-1 hairy roots.
